# General practice managers’ motivations for skill mix change in primary care: Results from a cross-sectional survey in England

**DOI:** 10.1177/13558196221117647

**Published:** 2022-08-17

**Authors:** Jon Gibson, Anne McBride, Katherine Checkland, Mhorag Goff, Mark Hann, Damian Hodgson, Imelda McDermott, Matt Sutton, Sharon Spooner

**Affiliations:** 1 Research Fellow, Centre for Primary Care and Health Services Research, 5292 University of Manchester, UK; 2 Professor of Employment Relations, Alliance Manchester Business School, 5292 University of Manchester, UK; 3 Professor of Health Policy & Primary Care, Centre for Primary Care and Health Services Research, 5292 University of Manchester, UK; 4 Research Associate, Centre for Pharmacy Workforce Studies, 5292 University of Manchester, UK; 5 Senior Research Fellow, Centre for Primary Care and Health Services Research, 5292 University of Manchester, UK; 6 Professor of Organisational Studies, Sheffield University Management School, 7315 University of Sheffield, UK; 7 Research Fellow, Centre for Primary Care and Health Services Research, 5292 University of Manchester, UK; 8 Chair in Health Economics, Centre for Primary Care and Health Services Research, 5292 University of Manchester, UK; 9 Clinical Lecturer, Centre for Primary Care and Health Services Research, 5292 University of Manchester, UK

## Abstract

**Objectives:**

The objectives are to determine the factors that motivated GP practice managers in England to employ non-medical roles, and to identify an ideal hypothetical GP practice workforce.

**Methods:**

Cross-sectional survey of GP practice managers in England (*n* = 1205). The survey focused on six non-medical roles: advanced nurse practitioner, specialist nurse, health care assistant, physician associate, paramedic and pharmacist.

**Results:**

The three most commonly selected motivating factors were: (i) to achieve a better match between what patients need and what the practitioner team can deliver; (ii) to increase overall appointment availability and (iii) to release GP time. Employment of pharmacists and physician associates was most commonly supported by additional funding. Practice managers preferred accessing new non-medical roles through a primary care network or similar, while there was a clear preference for direct employment of additional GPs, advanced nurse practitioners or practice nurses. The ideal practice workforce would comprise over 70% of GPs and nurses, containing, on average, fewer GPs than the current GP practice workforce.

**Conclusion:**

This study confirms that more diverse teams of practitioners are playing an increasing role in providing primary care in England. Managers prefer not to employ all new roles directly within the practice. A more detailed investigation of future workforce requirements is necessary to ensure that health policy supports the funding (whether practice or population based), recruitment, training, deployment and workloads associated with the mix of roles needed in an effective primary care workforce.

## Introduction

Many countries are experiencing significant problems with the recruitment and retention of physicians in primary care.^1–3^ In England, as a result of workforce shortages and the increasing frailty and complexity of patients, general practitioners (GPs) have seen substantial increases in their workload.^4^ This has implications for patients needing timely access to primary and secondary care,^5,6^ and exacerbates retention of GPs and wider workforce challenges.^7^

Skill mix change has been proposed as a way to address these challenges, that is, expanding the primary care workforce by employing a diverse range of practitioners to free up GP time for more complex patients, offer additional health care services or act as partial or total substitutes for GPs.^8,9^ However, any reconfiguration of the primary care workforce will require active management to ensure it does not undermine continuity of care or lead to reduced productivity.^10^

For example, there is some evidence that adding nursing staff does not always release doctor time and instead can create a duplication of appointments or workload.^10–12^

In 2016, the government in England launched a strategy to strengthen primary care, which included plans to expand the non-medical primary care workforce.^13^ It was followed by funding for an additional 26,000 primary care staff through newly developed primary care networks (PCNs),^14^ whereby groups of neighbouring GP practices can obtain limited funding to employ additional non-medical staff under the ‘Additional Roles Reimbursement Scheme’ (ARRS). Funding was initially restricted to a small number of roles, such as clinical pharmacists, physician associates, physiotherapists and paramedics, but was later broadened to include other roles such as dieticians and podiatrists.

The potential for the ARRS to accelerate workforce change in primary care will be influenced by whether, and how, practice workforce deployment is driven by or responsive to changes in health policy. Relatively little is known about why providers choose particular approaches to skill mix or the context in which these decisions are made.^15,16^ Among the range of possible reasons for employing non-medical staff is the availability of funding.^17^ While funding provides an initial catalyst, if changes in the workforce are primarily motivated by financial incentives, this could undermine the sustainability and long-term embeddedness of employing non-medical staff where initial funding support is not maintained.^18^

In this paper, we present the findings from a survey of GP practice managers in England. It sought to investigate the factors that motivated GP practices’ decisions to employ new roles and the role that financial incentives played in decision making. It further explored future intentions to do so as well as practice managers’ ideal hypothetical workforce, thereby providing information that can inform training and workforce upskilling programmes.

## Methods

### Survey administration

We developed an online survey targeting GP practice managers because they are responsible for informing NHS England (national body overseeing the budget, planning and delivery of the National Health Service (NHS) in England) about the composition of their workforce, and are likely to have the most up to date knowledge about workforce composition and plans for workforce expansion.

All GP practices in England were eligible to participate in the study. Invitations to participate were distributed using a link sent by email from a practice’s Local Clinical Research Network (LCRN). However, not all practices will have received an invitation as they may have opted out of the research mailing lists. Practices were incentivised to respond through the prospect of inclusion in a random prize draw with four prizes of £250.

The survey was conducted between August 2019 and December 2019, with LCRNs asked to send invitations on three occasions to maximise recruitment. The online questionnaire was created using Sawtooth Software’s Lighthouse Studio (version 9.7.2) and was hosted on University of Manchester servers.

### Survey content

The study focused on six roles: advanced nurse practitioner, specialist nurse, health care assistant, physician associate, paramedic and pharmacist. These represent a mix of staff whose employment in GP practices either largely predates the ARRS scheme, have been linked to financial incentives from earlier regional and national policy, offer additional (new) services or are additional roles that can *also* be funded through PCNs.

We developed two sets of questions. The first set asked practices who employed one of the above six roles about the factors that had influenced their decision to employ staff in that role. Respondents were presented with a list of 12 predefined factors, which we had identified from the literature and previous workforce research. Selection of these factors was informed by a review of descriptions of the aspects of health care that different types of practitioners can provide,^19^ and by changes in health policy^13^ and approaches to service delivery in professional discourses^20^ and refined by discussion and piloting with GPs and practice managers. Respondents were invited to add factors not captured in the list using free-text. Practice managers were asked to select all factors that applied to their decision for each type of worker. They were then asked if they had received funding specifically to support employment of these staff, and, if so, to name the funding organisation, and whether they were still receiving funding.

The second set of questions was posed to all GP practices regardless of whether they currently employed any of the six roles of interest. Practice managers were asked if they would in future wish to employ additional staff from a list of ten roles and, if so, whether they would prefer them to be directly employed by the practice or through a PCN. Finally, practices were asked to identify their ideal workforce composition by selecting the percentage of their total clinical workforce that would be made up of each of the ten listed roles. GPs were included in the list of roles for this question. Respondents were asked to use slider bars to indicate the percentages for each worker group. These bars were programmed so that total percentages automatically adjusted to 100%.

### Data analysis

We present statistics for the responses to the questions that asked about financial support, desire for additional roles and ideal workforce. These are the percentage or proportion of practices selecting each option. For continuous variables, we present the mean and standard deviation. Factors that affected the likelihood of a practice being invited to participate or responding to the questionnaire could introduce bias if the characteristics and responses of the responding practices are systematically different from practices that were not part of our sample. We therefore used inverse probability weighting to reduce this potential for bias. To calculate these weights, we estimated a logistic regression model for the binary variable of whether a practice responded, using workforce, region and registered population characteristics as explanatory variables. These data were obtained from NHS Digital General Practice Workforce statistics.^21^ We predicted the probability of responding for each practice using this regression model and took the reciprocal of this fitted value as a weight. The results from the logistic regression are included in the Online Supplement. Data analysis was conducted using Stata 15.1.

## Results

### Response and sample

Survey responses were submitted by 1261 practice managers. Due to missing data from the practice completing the survey and from the external practice data used for weighting, 1205 complete practice responses (96% of 1261) were used for this analysis. Our sample accounted for around 17% of a total population of 7012 GP practices as at December 2019; it contained at least one respondent from 174 of the 191 (91%) Clinical Commissioning Groups (CCGs; groups of general practices that commission most of the hospital and community NHS services in the local areas for which they are responsible).

Table 1 presents practice characteristics from the practices in our sample alongside the same characteristics from all GP practices in England. We report data from 1205 responding practices and 5242 non-sample practices as not all characteristics were available for all GP practices (whether they responded or not).

**Table 1.** Sample characteristics and workforce full-time equivalents (FTE).

	Sample GP practices					Non-responding GP practices					Difference in means	
	Mean (M_S_)	Standard deviation	Median	*N*		Mean (M_NR_)	Standard deviation	Median	*N*		Difference (M_S_-M_NR_)	*p*-value
GP staff FTE	5.94	4.22	5.13	1205		4.84	3.62	4.01	5242		1.10	<0.001
Nurse staff FTE	2.98	2.86	2.25	1181		2.43	2.24	1.84	5123		0.54	<0.001
Direct patient care FTE	2.43	2.86	1.60	1205		1.82	2.31	1.05	5242		0.62	<0.001
Administration staff FTE	11.79	9.62	9.58	1204		9.87	7.39	8.27	5240		1.92	<0.001
Patient list size	10219.09	6897.27	8784.00	1205		8702.09	5669.90	7705.50	5242		1517.00	<0.001
% of patients living in urban areas	0.80	0.34	0.99	1205		0.83	0.33	0.99	5242		-0.29	0.006
Average patient income deprivation	0.13	0.06	0.11	1205		0.14	0.07	0.13	5242		-0.1	<0.001
Workforce FTE	Mean (M_S_)	Standard deviation	Median	*N*	Number of Practices with some staff [FTE >0] (%)	Mean (M_NR_)	Standard deviation	Median	*N*	# Practices FTE >0 (%)	Difference (M_S_-M_NR_)	*p*-value
Partner GP	3.10	2.34	2.69	1205	1118 (92.8)	2.60	1.98	2.13	5242	4878 (93.1)	0.50	<0.001
Salaried GP	1.55	1.87	1.07	1205	940 (78.0)	1.23	1.51	0.80	5242	3586 (68.4)	0.32	<0.001
Advanced nurse practitioner	0.66	1.01	0.00	1181	553 (45.9)	0.56	0.99	0.00	5123	2085 (39.8)	0.10	0.002
Specialist nurse	0.09	0.42	0.00	1181	110 (9.1)	0.07	0.33	0.00	5123	401 (7.6)	0.02	0.068
Practice nurse	2.03	1.88	1.60	1181	1142 (94.8)	1.66	1.37	1.36	5123	4953 (94.5)	0.37	<0.001
Health care assistant	1.20	1.23	0.96	1205	1005 (83.4)	0.94	1.02	0.77	5242	3934 (75.0)	0.26	<0.001
Physician associate	0.05	0.34	0.00	1205	48 (4.0)	0.04	0.25	0.00	5242	159 (3.0)	0.02	0.047
Pharmacist	0.26	0.61	0.00	1205	321 (26.6)	0.17	0.44	0.00	5242	1069 (20.4)	0.09	<0.001
Paramedic	0.12	0.43	0.00	1205	114 (9.5)	0.09	0.39	0.00	5242	357 (6.8)	0.03	0.017
Physiotherapist	0.01	0.08	0.00	1205	15 (1.2)	0.01	0.09	0.00	5242	60 (1.1)	0.00	0.911
Regions	Practices	Percentage				Practices	Percentage					
East of England	157	13.03				506	9.65					
London	303	25.15				838	15.99					
Midlands	166	13.78				1,102	21.02					
North East and Yorkshire	186	15.44				821	15.66					
North West	128	10.62				819	15.62					
South East of England	129	10.71				713	13.6					
South West of England	136	11.29				443	8.45					

Direct Patient Care is all other staff who are involved in patient care and are not classified as either GPs or nurses. Income deprivation is measured as the proportion of people living within an area receiving support for low income.

Practices in our sample tended to be larger than the non-sample practices in terms of workforce and patient list size. Mean GP full time equivalent (FTE) for the sample practices was 5.94 compared to a mean of 4.84 FTE for non-sample practices, a difference of 1.1 GP FTE. Sample practices also employed more FTEs across three workforce groups: nurses, direct patient care and administrative staff. The direct patient care group includes all other staff that provide care and who are not a GP or nurse, namely, pharmacists, physiotherapists and physician associates. Sample practice patient list sizes had a mean of 10,219 patients compared to a mean of 8702 among non-sample practices; a difference of 1517 patients. GPs based in more deprived locations were less likely to be part of the sample. The results from the logistic regression and the distribution of the resulting weights are shown in the Online Supplement.

### Motivating factors

**Figure 1.**
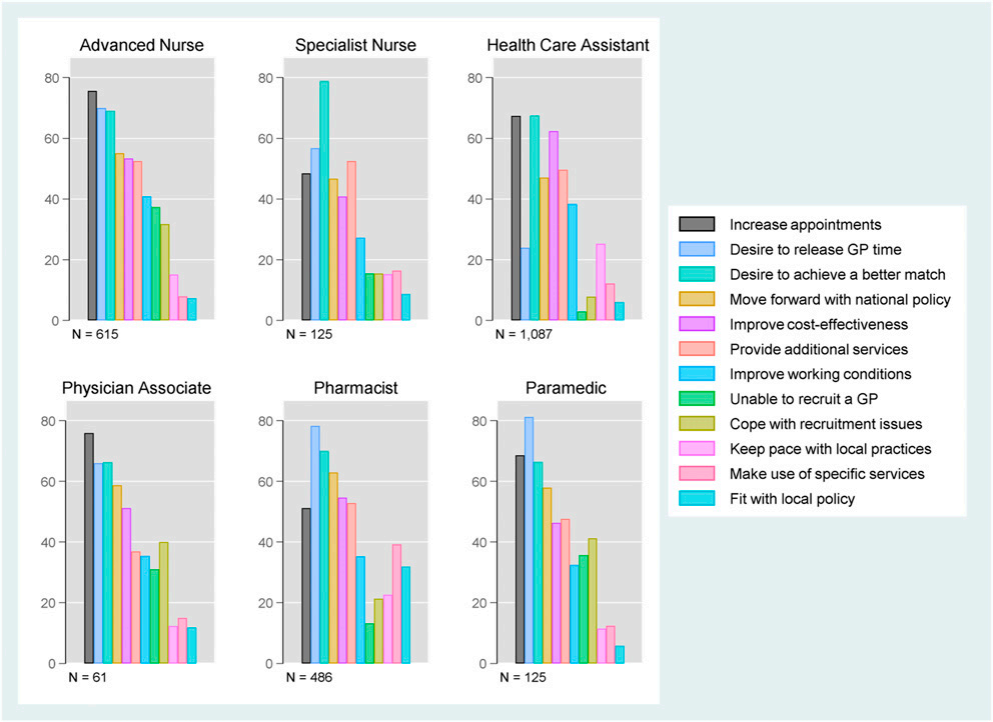
Motivating factors. Percentage of responding practices that are currently employing the role selecting motivating factors for employment of the role. Note: Responses are weighted by inverse probability weights (Online Supplement Table S1).

Figure 1 presents the proportion of GP practices that employed at least one of six roles and selected motivating factor for doing so at the time of the survey. The most commonly selected motivating factor for employing advanced nurse practitioners and physician associates was ‘to increase overall appointment availability’; for employing pharmacists and paramedics it was ‘desire to release GP time’ and for employing specialist nurses and health care assistants it was ‘desire to achieve a better match between what patients need and what the practitioner team can deliver’ (see also Online Supplement, Table S2). Supply factors such as ‘to cope with recruitment issues – our choices are limited by the availability of suitable practitioners’ and ‘unable to recruit a GP’ were most commonly selected by practices employing advanced nurse practitioners, paramedics and physician associates. Other commonly selected motivating factors across the staff groups were desire to ‘improve cost-effectiveness’, ‘move forward with national policy for skill mix (i.e. different types of practitioners)’ and ‘to provide additional or improved services to patients such as increased access beyond what is currently available’.

### External funding

Table 2 shows the percentages of practices that received external funding to support the employment of staff in the six roles of interest. The most commonly supported roles were pharmacists and physician associates, with 63% and 34% of practices reporting to employ these roles. Of the 486 practices that employed pharmacists, 31% reported receiving funding from NHS England and 18% reported receiving funding from local health care providers. Of the 61 practices that employed physician associates, 19% reported having received funding from Health Education England (the national leadership organisation for education, training and workforce development in the health sector) and 10% from their CCG. Around 16% of practices that employed paramedics and specialist nurses reported having received additional funding to support their employment. Just under one-quarter (24%) of practices that employed pharmacists reported that, at the time of completing the survey, they were still receiving additional funding.

**Table 2.** Percentage of GP practices that have received financial support to employ particular roles.

*N*=1205	Advanced nurse	Specialist nurse	Health care assistant	Physician associate	Pharmacist	Paramedic
	*N* = 615 (%)	*N* = 125 (%)	*N* = 1087 (%)	*N* = 61 (%)	*N* = 486 (%)	*N* = 125 (%)
None	73.7	64.1	72.4	45.8	27.4	72.6
Clinical commissioning group	3.5	11.0	3.5	9.8	15.0	7.8
Local network or federation	1.7	1.6	1.5	0.7	18.2	4.6
Health education England	3.2	1.8	2.8	18.7	3.3	1.4
NHS England	0.8	0.0	0.7	3.3	31.0	0.3
Other	1.0	2.0	0.8	5.2	2.7	2.9
Any financial incentive?	7.9	16.4	7.6	34.2	63.4	16.7
Don’t know	5.7	5.4	5.6	6.6	4.7	3.0
Still receive support?	1.9	4.4	1.5	14.9	23.6	5.7

Responses are weighted by inverse probability weights (Online Supplement Table S1).

### Desire for future access to additional roles

Table 3 shows the number of practices who indicated that they would like to have access to additional roles, either through direct employment or through a PCN or a GP federation. The most commonly desired roles were the more traditional roles: salaried GPs, advanced nurse practitioners, GP partners and practice nurses. Of the practices that indicated that they desired more staff from new primary care roles, including physician associate, pharmacists, paramedics or physiotherapists, the majority indicated that they wished to access these through a network, federation or other existing organisation. Over half (57.7%) of practices indicated that they desired access to physiotherapists from outside the practice and 25.9% of practices indicated that they would like access to additional physician associates in the same way.

**Table 3.** Percentage of GP practices that would like access to additional roles.

*N* = 1205	Employed through our own practice, %	Access through a network, federation or similar organisation, %
Salaried GP	41.3	14.0
Advanced nurse practitioner	33.2	21.6
Partner GP	32.0	3.5
Practice nurse	28.3	10.0
Pharmacist	23.5	53.3
Healthcare assistant	22.6	9.7
Physiotherapist	16.0	57.7
Paramedic	14.3	45.2
Physician associate	14.0	25.9
Specialist nurse	11.5	25.9

Responses are weighted by inverse probability weights (Online Supplement Table S1).

### Ideal workforce

**Figure 2.**
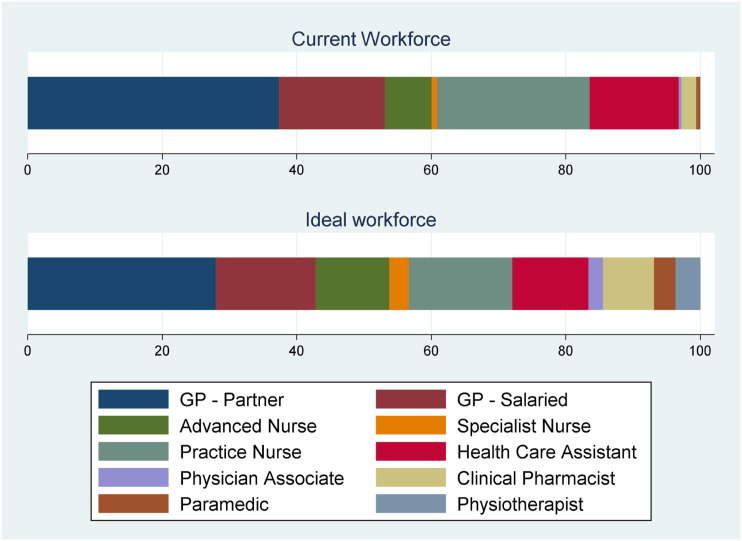
Current and ideal workforce composition (%). Note: Responses are weighted by inverse probability weights (Online Supplement Table S1). *N* = 880.

Figure 2 and Table 4 present the findings about the ideal workforce that practice managers indicated in their survey responses. In Table 4, we present the percentage of practices selecting the staff role to be in their ideal workforce and the mean percentage that the role should form of the ideal hypothetical workforce. The most commonly selected roles were GP partners (selected by 89.9% of responding practices and constituting 28% of the mean ideal workforce) and salaried GPs (comprising 15% of the mean ideal workforce), with practice nurses next (15.4%), followed by advanced nurse practitioners (10.9%) and health care assistants (11.3%).

**Table 4.** Comparison of current and stated ideal workforce.

	Current workforce			Ideal workforce			Correlation between means	*p*-value
*N* = 880	% practices with some of these staff already in workforce (FTE >0)	Mean % of workforce (FTE)	SD	% practices selecting some of these staff (>0%)	Mean % of ideal workforce	SD		
Partner GP	92.4	37.3	19.59	89.8	28.1	19.28	0.34	<0.001
Salaried GP	73.3	15.7	15.28	76.8	14.8	12.94	0.39	<0.001
Advanced nurse practitioner	48.3	7.0	9.35	76.2	10.9	9.49	0.37	<0.001
Specialist nurse	10.0	0.8	3.32	36.5	2.9	5.22	0.17	<0.001
Practice nurse	96.6	22.7	10.83	90.0	15.4	8.87	0.12	<0.001
Health care assistant	83.1	13.2	9.58	87.3	11.3	8.31	0.23	<0.001
Physician associate	5.3	0.4	2.30	26.3	2.2	4.88	0.25	<0.001
Pharmacist	25.4	2.2	5.12	78.2	7.6	6.99	0.16	<0.001
Paramedic	8.1	0.6	2.45	45.4	3.2	4.39	0.35	<0.001
Physiotherapist	2.6	0.02	0.31	54.9	3.7	4.35	0.07	0.031

Among other roles, pharmacists were most commonly selected to be part of the workforce (78% of respondents) and formed 7.6% of the mean ideal workforce. This compares with 25% of the responding practices currently employing pharmacists. Physician associates were selected by 26% of respondents and formed 2.2% of the mean ideal workforce. New primary care workforce roles such as pharmacists, physiotherapists, physician associates and paramedics constituted a greater proportion of the ideal workforce relative to the current workforce employed by responding practices.

## Discussion

This study reports on a survey of GP practice managers’ preferences for staff roles and desired skill mix in England. The most common factors motivating practices to employ staff in non-GP roles were: (i) achieving a better match between what patients need and what the practitioner team can deliver, in particular through specialist nurses, (ii) increasing overall appointment availability (physician associates and advanced nurse practitioners) and (iii) releasing GP time (paramedics and pharmacists). Our analysis found that pharmacists and physician associates were most commonly supported by specific additional funding, although this funding was not reported to be a key motivating factor for their current employment. Practices interested in employing physician associates, pharmacists, paramedics or physiotherapists reported preferring to do this through a PCN, a federation or similar, while direct employment was preferred for any additional GPs, advanced nurse practitioners or practice nurses. The ideal practice workforce would comprise over 70% of GPs and nurses, containing, on average, fewer GPs than the current workforce. New roles comprised less than 20% of the ideal practice workforce, with pharmacists the most commonly selected role.

### Strengths and limitations

The study’s key strengths are in the collection of a large dataset on various aspects around skill mix in GP practices in England. We asked practice managers to complete the survey on behalf of their GP practice. Our survey does thus not capture preferences expressed by, for example, GP partners, their colleagues or patients. Our dataset allows us to compare practice managers’ ideal practice workforce composition with the profile of their current workforce as reported to NHS Digital. The response rate was low, at 17%. We have used weighting to minimise response bias in the sample. Response bias may remain if responding and non-responding practices differ by factors that we were unable to observe in our study.

### Comparison with existing literature

This study confirms that more diverse teams of practitioners are playing an increasing role in providing primary care in England. Practices appear to be addressing three main issues in changing their skill mix^22^: (i) How do we better match what patients’ need? (ii) How do we increase overall appointment availability? (iii) How do we release GP time? These reasons have resonance with doctors and nurses who see nurse-doctor substitution as a way of increasing patient access to care^23^ and discussions about how GP time could potentially be released to better match skills to disease.^24^ It adds to existing studies that have focused on primary care workforce deployment decisions in relation to specific practitioner types.^25^

Additional funding to encourage new ways of working can incentivise skill mix change,^17^ but there is a risk that if additional funding for new roles is not maintained, then new roles may cease with the funding.^18^ Our study points to some evidence that this outcome is less likely to be the case here. First, responding practice managers did not appear to prioritise funding and incentives as the key motivating factors for appointing non-medical roles. Second, with the exception of pharmacists, and, to a much lesser extent physician associates, the majority of practices already employed advanced nurse practitioners, specialist nurses, health care assistants, or paramedics without incentives. Third, physician associates, pharmacists, paramedics and physiotherapists were included in the ideal practice workforce. However, managers did express a preference for not directly employing a number of new roles. Without careful attention from managers (in practices and primary care networks), skill mix changes continually enabled through external resources might lead to service improvements at greater overall cost.^26^

## Conclusions

Managers are prioritising the addressing of three different issues through skill mix change (achieving a better match of demand and supply; increasing appointment availability and releasing GP time). The principal motivations driving employment of non-medical staff differed between types of role. Managers preferred to employ some roles within their own organisation but preferred other roles to be externally provided.

The extent to which GP practice managers’ ideal future workforce differed from the current workforce highlights a need for more detailed investigation of future workforce requirements to ensure that health policy supports the funding (whether practice or population based), recruitment, training, deployment and workloads associated with the mix of roles needed and desired in an effective primary care workforce.

## Supplemental Material

Supplemental Material - General practice managers’ motivations for skill mix change in primary care: Results from a cross-sectional survey in England Click here for additional data file. Supplemental Material for General practice managers’ motivations for skill mix change in primary care: Results from a cross-sectional survey in England by Jon Gibson, Anne McBride, Katherine Checkland, Mhorag Goff, Mark Hann, Damian Hodgson, Imelda McDermott, Matt Sutton and Sharon Spooner in Journal of Health Services Research & Policy
