# Supplementary material for: General practice managers’ motivations for skill mix change in primary care: Results from a cross-sectional survey in England
Source: J Health Serv Res Policy. 2022 Aug 17;28(1):5–13. doi: 10.1177/13558196221117647 (PMC9850398; doi:10.1177/13558196221117647)

Online Supplement

Table S1: Logistic regression: Response to survey

Response (1 = yes, 0 = no)	Coefficient.	Std. Err.	[95% Conf. Interval]	
Practice GP FTE	0.05	0.01	0.03	0.07
Practice Direct Patient Care FTE	0.05	0.01	0.03	0.08
Income Deprivation	-2.34	0.56	-3.45	-1.24
**Regions**				
London	0.40	0.12	0.17	0.63
Midlands	-0.59	0.13	-0.84	-0.34
North East and Yorkshire	-0.17	0.13	-0.41	0.08
North West	-0.42	0.14	-0.69	-0.15
South East of England	-0.58	0.13	-0.84	-0.31
South West of England	-0.08	0.14	-0.34	0.19
Constant	-1.36	0.12	-1.60	-1.12
N	6447			
Pseudo R2	0.04			
Log likelihood	-2991.24			

Note: FTE is the number of practitioner type full time equivalents employed at the practice.

Table S2. Motivating factors: Percent of GP practices with practitioner type who selected motivating factor

	Practices who employed					
	Advanced Nurse Practitioner N=615	Specialist Nurse N=125	Health Care Assistant N=1087	Physician Associate N=61	Pharmacist N=486	Paramedic N=125
To increase overall appointment availability	75.58%	48.43%	67.38%	75.90%	51.20%	68.58%
Desire to release GP time	69.97%	56.74%	23.94%	65.95%	78.27%	81.25%
Desire to achieve a better match between what patients need and what the practitioner team can deliver	69.04%	78.92%	67.48%	66.36%	70.02%	66.42%
Desire to move forward with national policy for skill mix (i.e. different types of practitioners)	55.13%	46.67%	47.04%	58.72%	62.92%	57.92%
Desire to improve cost-effectiveness	53.37%	40.81%	62.37%	51.19%	54.62%	46.31%
To provide additional or improved services to patients such as increased access beyond what is currently available	52.46%	52.49%	49.62%	36.90%	52.83%	47.64%
Desire to improve working conditions for practice staff	40.79%	27.21%	38.35%	35.42%	35.30%	32.45%
Unable to recruit a GP	37.40%	15.50%	2.90%	31.00%	13.20%	35.70%
To cope with recruitment issues – our choices are limited by the availability of suitable practitioners	31.71%	15.44%	7.81%	40.03%	21.33%	41.21%
To keep pace with the range of services offered by other local practices	15.09%	15.19%	25.23%	12.29%	22.62%	11.49%
To make use of specific services, funding or incentives provided a local CCG, federation or GP network etc.	7.90%	16.41%	12.18%	14.98%	39.26%	12.45%
To fit with local policy decisions e.g. such as funding for specific types of practitioner through an incentive scheme	7.29%	8.72%	6.05%	11.79%	31.90%	5.80%

Note: Responses are weighted by inverse probability weights from the regression

Figure S1: Histogram of inverse probability weights

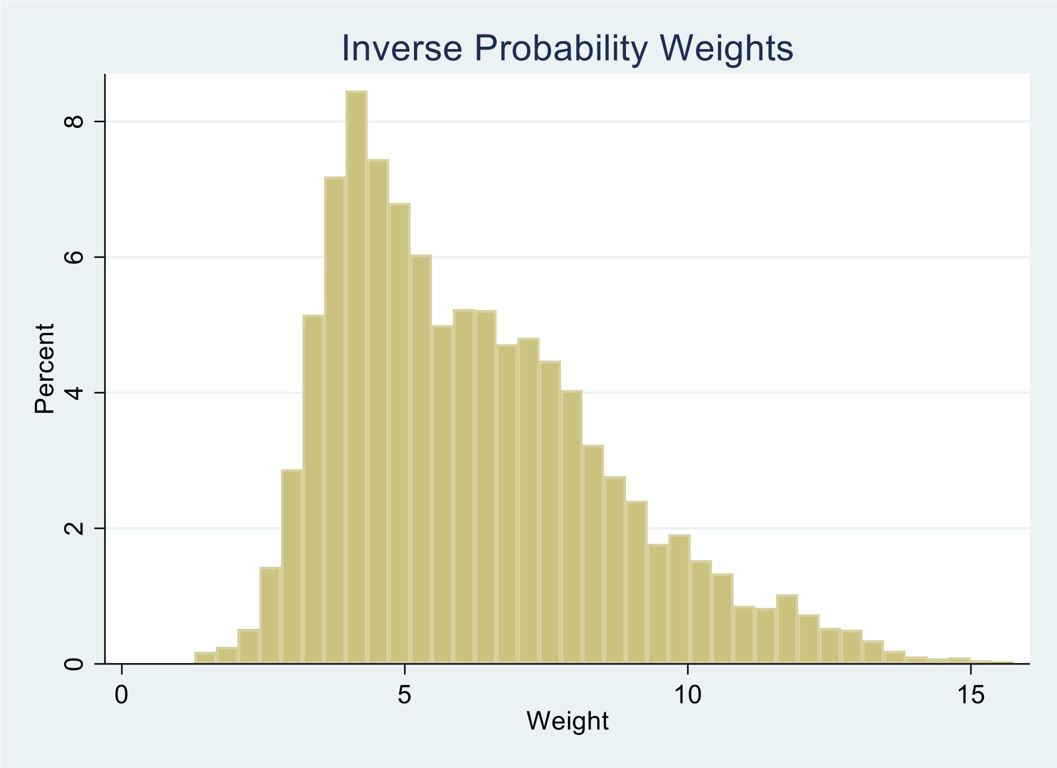

Supplement: Supplemental Material - General practice managers’ motivations for skill mix change in primary care: Results from a cross-sectional survey in England [file sj-pdf-1-hsr-10.1177_13558196221117647.pdf]
